# Optimizing Vegetation Configurations for Seasonal Thermal Comfort in Campus Courtyards: An ENVI-Met Study in Hot Summer and Cold Winter Climates

**DOI:** 10.3390/plants14111670

**Published:** 2025-05-30

**Authors:** Hailu Qin, Bailing Zhou

**Affiliations:** School of Urban Construction, Wuhan University of Science and Technology, Wuhan 430065, China; 202108406054@wust.edu.cn

**Keywords:** outdoor thermal comfort, vegetation configuration, ENVI-met simulation, subtropical monsoon climate, courtyard microclimate, physiological equivalent temperature

## Abstract

This study investigated the synergistic effects of vegetation configurations and microclimate factors on seasonal thermal comfort in a semi-enclosed university courtyard in Wuhan, located in China’s Hot Summer and Cold Winter climate zone (Köppen: Cfa, humid subtropical). By adopting a field measurement–simulation–validation framework, spatial parameters and annual microclimate data were collected using laser distance meters and multifunctional environmental sensors. A validated ENVI-met model (grid resolution: 2 m × 2 m × 2 m, verified by field measurements for microclimate parameters) simulated 15 vegetation scenarios with varying planting patterns, evergreen–deciduous ratios (0–100%), and ground covers. The Physiological Equivalent Temperature (PET) index quantified thermal comfort improvements relative to the baseline. The optimal grid-based mixed planting configuration (40% evergreen trees + 60% deciduous trees) significantly improved winter thermal comfort by raising the PET from 9.24 °C to 15.42 °C (66.98% increase) through windbreak effects while maintaining summer thermal stability with only a 1.94% PET increase (34.60 °C to 35.27 °C) via enhanced transpiration and airflow regulation. This study provides actionable guidelines for climate-responsive courtyard design, emphasizing adaptive vegetation ratios and spatial geometry alignment.

## 1. Introduction

### 1.1. Background

Under the combined pressures of climate change and rapid urbanization, campus environments—as educational spaces with high population density—directly influence the health and well-being of students through their outdoor thermal comfort, thereby affecting the sustainability of pedagogical activities [1]. In the Hot Summer and Cold Winter (HSCW) climate zone of central China (e.g., Wuhan, Köppen Cfa), extreme seasonal temperature variations pose dual challenges: oppressive heat and humidity during summer months (July mean >28 °C) and cold winds with low temperatures in winter (January mean 3–5 °C). These conditions render conventional greening strategies inadequate for year-round thermal environment regulation.

While existing studies have predominantly focused on single-season analyses or specific climate types [2,3,4], there has been limited exploration into the dynamic thermal comfort mechanisms across transitional climate zones [5]. Campus courtyards, as semi-open spaces integrating social interaction and leisure functions, require urgent quantification of the coupling effects between vegetation layouts and microclimate modulation.

### 1.2. Literature Review and Research Gap

Research on campus vegetation configurations and thermal comfort mechanisms necessitates an in-depth exploration of their spatial heterogeneity and ecological response dynamics. From a functional zoning perspective, academic areas often utilize low-canopy trees to create semi-transparent covers that partially filter solar radiation while maintaining indoor daylight levels, thereby achieving a dynamic balance with classroom lighting requirements [5]. In contrast, residential zones prioritize vertical greening strategies with greater thermodynamic optimization potential: climbing vegetation on building façades has been shown to reduce west-facing wall surface temperatures by 2.1–4.6 °C while enhancing air humidity through foliar transpiration [6]. Research also reveals significant temporal variations in courtyard thermal comfort. For instance, ENVI-met simulations of semi-outdoor spaces (e.g., elevated plazas and courtyards) in Guangdong’s hot–humid climate show that the afternoon Universal Thermal Climate Index at 14:00 exceeded morning (08:00) levels by 1.8–2.5 °C due to solar radiation dynamics [7]. Observations at Assiut University further demonstrated that PET exceedance rates reached 42% during building occupancy periods versus 12% post-closure [8], indicating the need for usage–intensity-adjusted comfort criteria.

Nevertheless, current research predominantly examines single-season effects, such as summer shading [7,9] or winter windbreak [10], without establishing cross-seasonal synergy models [5]. A representative case in Japan’s Hokuriku region illustrates this gap: evergreen shrubs effectively lower winter WS by 18–23%, yet their dense canopies concurrently block solar radiation, resulting in summer predicted mean vote (PMV) values exceeding other configurations by 0.8–1.2 units [11]. Such trade-offs between thermal gain mitigation and wind protection efficacy remain systematically unquantified.

Thermal environment regulation in HSCW climate zones involves navigating dynamic seasonal contradictions, requiring interdisciplinary frameworks that harmonize vegetation functionality, spatial layouts, and climatic responsiveness. During summer, tall deciduous trees serve as primary thermal comfort enhancers through shading and evapotranspiration, though excessive planting densities may impede airflow velocities by 30–45% [12]. Composite designs integrating climbing plants (e.g., ivy) with trees demonstrate synergistic benefits: vertical greening reduces façade temperatures without compromising cross-ventilation efficiency. A Hangzhou, China, case study reported a maximum Physiological Equivalent Temperature (PET) reduction of 27.9 °C under such configurations, with no significant airflow obstruction observed [13]. Winter conditions introduce conflicting priorities, where evergreen vegetation’s windbreak efficiency clashes with solar access needs. Research confirms that evergreen canopies block 60–75% of winter radiation, inducing cold stress with PET decreases up to 3.57 °C [12]. Transitional seasons (10–25 °C) further complicate thermal regulation, as existing vegetation designs persist with static summer/winter templates despite the nonlinear thermal demand characteristic of spring and autumn.

ENVI-met, a benchmark tool in urban microclimate simulation, has undergone transformative advancements from single-physics modeling to multi-physics coupling capabilities. Initially released in 1998 by Michael Bruse’s team, its early versions focused on building–surface thermal exchanges using simplified 2D vegetation models [2]. Post-2010 updates integrated plant physiological processes (e.g., stomatal conductance) and human thermal comfort indices (PET and UTCI), culminating in the v4.0 release that introduced 3D plant databases parameterized by the leaf area index (LAI) and density (LAD), enabling radiation interception modeling at individual tree scales [14]. The embedded BIO-met module now quantifies real-time interactions between plant transpiration and thermal comfort metrics, with recent studies revealing ±1.5–2.8 °C cooling variations under identical green space ratios due to species-specific transpiration rates [15].

Compared to alternatives, ENVI-met’s strengths lie in three domains: (1) bidirectional vegetation–atmosphere feedbacks, dynamically linking stomatal responses to humidity fields, a capability absent in Fluent’s turbulence-centric approach [16]; (2) sub-meter spatial resolution critical for resolving tree shading effects, surpassing EnergyPlus’s 10 m-scale limitations [16]; and (3) cross-seasonal adaptability through its Tree Calendar function, which synchronizes leaf area decay with radiative transfer, outperforming PHOENICS’s reliance on preset phenology schedules [6]. The relevant literature is summarized in Table 1.

Current research exhibits three critical limitations. First, most studies adopt static analyses of single-season scenarios, such as summer cooling or winter insulation, while neglecting systemic investigations into year-round dynamic thermal comfort regulation mechanisms. Second, prevailing vegetation strategies inadequately address seasonal contradictions: evergreen species improve winter wind protection yet exacerbate cold stress (reducing PET by up to 3.57 °C in solar-deprived zones), while deciduous species’ phenological impacts on transitional-season thermal neutrality remain unquantified. Additionally, interdisciplinary integration is underdeveloped, particularly in coupling spatial morphology metrics (e.g., sky view factor and aspect ratio) with vegetation configuration optimization.

### 1.3. Purpose of This Work

Building upon this context, this study aims to resolve seasonal trade-offs via multi-scale modeling and empirical validation, establishing a cross-seasonal synergy framework for outdoor thermal environments in HSCW climate campuses. The specific objectives include (1) developing a multi-physics ENVI-met model that integrates 3D plant databases and BIO-met thermal comfort modules to simulate coupled radiation–ventilation–humidity interactions from individual trees to courtyard scales, (2) proposing layered greening designs for academic zones that balance daylighting and shading across seasons, (3) quantifying transitional-season thermal neutrality mechanisms through parametric vegetation layouts, and (4) formulating climate-responsive design guidelines with zone- and season-specific vegetation standards. By bridging gaps in transitional climate research, this work advances campus thermal management from a static design toward lifecycle-adaptive paradigms.

## 2. Methodology

**Figure 1:**
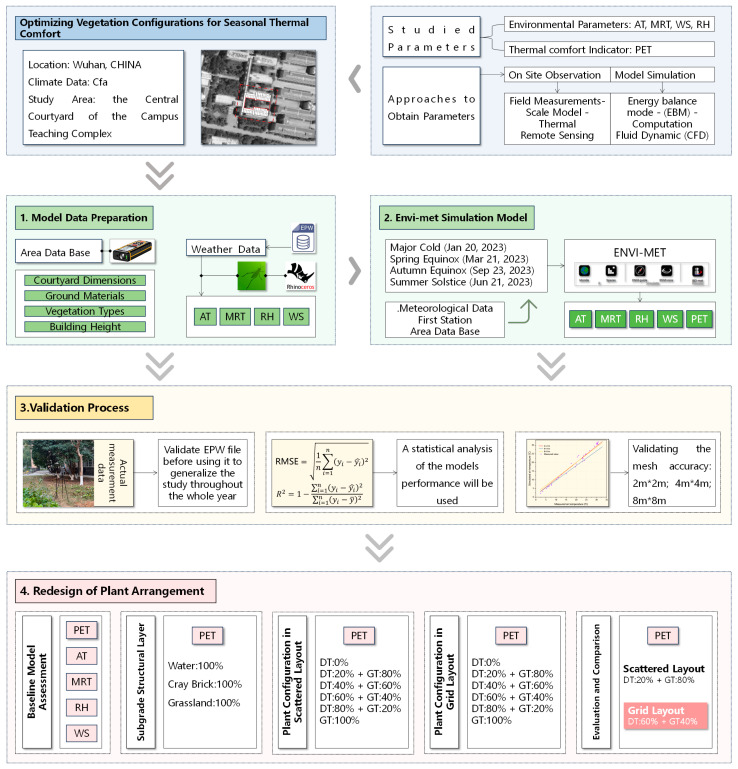
Workflow of this research.

This study focused on a typical courtyard enclosed by a standard teaching building in a university in Wuhan. It employed a “measurement-simulation-validation” research framework. Initially, we continuously monitored and obtained the spatial form parameters and annual microclimate data of the courtyard using a laser rangefinder and a multifunctional instrument. Based on the measured data, we constructed a benchmark ENVI-met model and validated its reliability through annual temperature field verification and grid precision testing. On this basis, we established 15 vegetation configuration experimental scenarios. The controlled variables included (1) planting patterns (random and grid), (2) the proportion of evergreen and deciduous trees (ranging from 0:100 to 100:0), and (3) ground cover types (full lawn, hard paving, and water body composite). While tree spacing was fixed in grid layouts (4.2 × 4.0 m), random distributions created localized density variations that may influence microclimate gradients at sub-courtyard scales—a potential focus for future high-resolution studies. All scenarios were compared with the existing courtyard as the baseline. The PET index was used to quantitatively evaluate the thermal environment improvement effects of each scheme. The specific technical route is shown in Figure 1.

### 2.1. Site and Data

**Figure 2:**
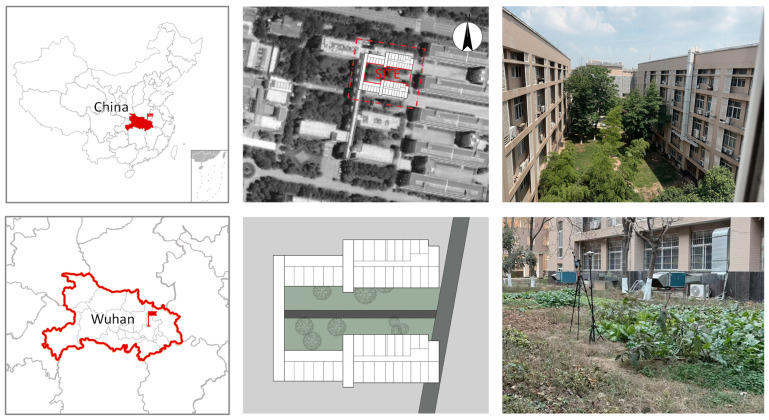
Location plan and on-site images.

As illustrated in Figure 2, the courtyard is enclosed by two five-story teaching buildings (20.3 m in height) to the north and south, with a connecting structure on the western side. The courtyard covers an area of 1162.55 m^2^ and contains nine scattered trees. The ground surface consists primarily of turf (988.25 m^2^, 85% coverage), with a central gray-brick pathway (174.3 m^2^, 15% coverage). Due to the irregular building facades, the courtyard’s edges exhibit non-uniform geometry.

Wuhan features a subtropical monsoon climate characterized by distinct seasonal variations, with hot, humid summers and cold, dry winters—typical of HSCW zones. The hottest month (August) records an average temperature of 31 °C, coupled with high humidity and frequent rainfall, significantly impacting outdoor thermal comfort. In contrast, winter months (December–February) experience average temperatures as low as 0 °C, with minimums reaching −5 °C, exacerbated by dry northerly winds that further deteriorate outdoor conditions [12]. This climatic duality necessitates a balanced approach to vegetation design, addressing both summer heat mitigation and winter cold stress—a challenge often overlooked in conventional studies focusing on single-season solutions.

The site’s microclimate is further influenced by its architectural geometry and ground cover composition, creating localized variations in solar exposure and wind patterns. Such conditions make it an ideal case for investigating vegetation strategies that optimize thermal comfort across contrasting seasonal demands, particularly in transitional periods where neither summer nor winter design principles fully apply.

### 2.2. ENVI-Met Set-Up

This study employed ENVI-met for numerical simulations, a computational fluid dynamic (CFD) and thermodynamics-based software that integrates the BIO-met module for the calculation of thermal comfort indices and the Albero tool for advanced vegetation modeling. These features enable high-resolution simulations of interactions among ground surfaces, vegetation, buildings, and atmospheric layers at micro-scales [17]. ENVI-met v5.7 incorporates refined vegetation modeling parameters, including canopy geometry, leaf area density (LAD), and root area density (RAD), to accurately capture tree transpiration effects and aerodynamic influences on wind flow patterns [18].

This study utilized standard EPW files sourced from Climate.OneBuilding.org for Wuhan; they contain validated hourly weather observations of temperature, humidity, and solar radiation. Following data acquisition, air temperature (AT) and relative humidity (RH) values were extracted using Ladybug Tools in Grasshopper before being converted to ENVI-met-compatible CSV format through custom Python 3.11 scripts that implemented quality control checks. The processed meteorological data were subsequently incorporated into ENVI-met’s intermediate simulation mode via the boundary condition editor, with temporal interpolation applied to maintain consistency between hourly measurements.

### 2.3. Model Validation

This study employed a multifunctional meteorological monitoring device (JT2020-3) for the continuous measurement of courtyard microclimate parameters. The instrument integrates high-precision sensors capable of synchronous measurements of multiple environmental indicators, including relative humidity (10–90% RH range, ±3% RH accuracy), air temperature (−20 °C to 85 °C range, ±0.5 °C accuracy), and air velocity (0.05–2.0 m/s range: ±[0.05 m/s + 2% reading]; 2.0–5.0 m/s range: ±[0.1 m/s + 2% reading]). The pyranometer features a 2π steradian measurement range with a 300–3200 nm spectral response (0–2000 W/m^2^ measurement range, 7–14 μV/(W/m^2^) sensitivity), demonstrating less than ±2% nonlinear error and ±2% temperature stability within −10 °C to +40 °C. The device also supports multidimensional calculation and the assessment of environmental thermal comfort. See Figure 2 for fieldwork photos.

**Figure 3:**
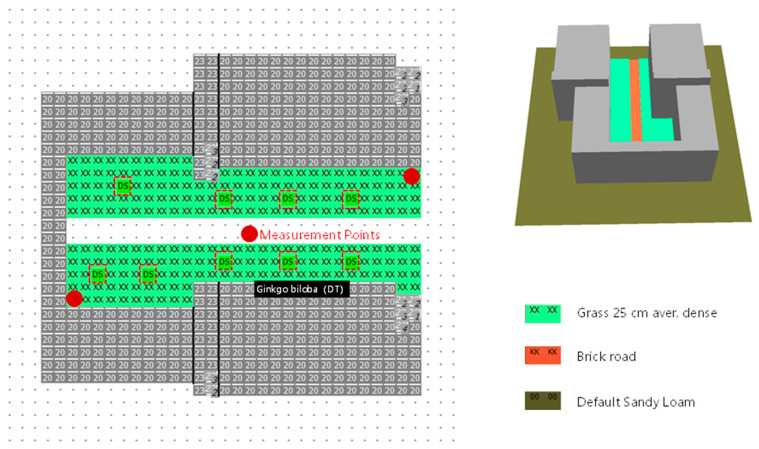
Envi-met model screenshots of the site.

To comprehensively characterize the microclimate conditions, three monitoring points were strategically positioned along the courtyard’s longitudinal diagonal at both endpoints and the midpoint (Figure 3). This triangular configuration effectively captures the spatial gradients of environmental parameters across the courtyard. Measurements were conducted three times monthly (on the 1st, 10th, and 20th) following a standardized diurnal monitoring protocol. During months containing solstices or equinoxes (e.g., winter solstice and spring equinox), additional 24 h continuous measurements were performed on these astronomically significant dates to obtain climatically representative data. The validation campaign specifically targeted the transitional periods between HSCW seasons to evaluate model performance under thermally challenging conditions.

A systematic comparison was conducted between field measurements and ENVI-met simulation results to evaluate model performance. The study area, covering 6400 m^2^, was simulated using three grid resolutions for comparative analysis: fine (2 × 2 m), medium (4 × 4 m), and coarse (8 × 8 m) grids. This multi-scale approach enabled the assessment of simulation accuracy across different spatial resolutions, providing a basis for selecting the optimal grid scale for subsequent analyses. The validation process ensured that simulation results reliably represented actual microclimate characteristics.

Air temperature (AT) was selected as the primary validation metric, consistent with established practices in similar studies [12,13,19]. Two statistical indicators were employed for quantitative evaluation: the coefficient of determination (R^2^) and root mean square error (RMSE). The coefficient of determination (R^2^), ranging from 0 to 1, quantifies the proportion of variance in observed data explained by the model, with values closer to 1 indicating a better fit. R^2^ was calculated as follows (Equation (1)) [20]:

(1) $$R^{2}=1-\frac{\sum_{i=1}^{n}{\left(y_{i}-{\hat{y}}_{i}\right)}^{2}}{\sum_{i=1}^{n}{\left(y_{i}-\bar{y}\right)}^{2}}$$

The RMSE, representing the square root of the mean squared error, measures the absolute difference between the predicted and observed values, where lower values indicate higher predictive accuracy. RMSE was computed using Equation (2) [20]:

(2) $$RMSE=\sqrt{\frac{1}{n}\sum_{i=1}^{n}{\left(y_{i}-{\hat{y}}_{i}\right)}^{2}}$$

**Figure 4:**
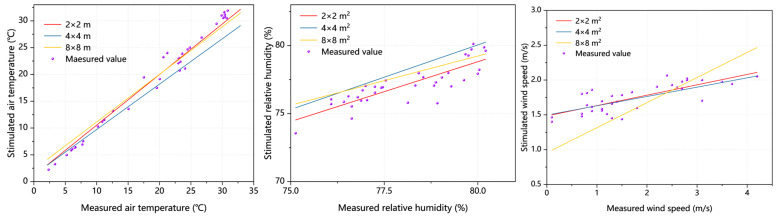
Validation results of coefficient of determination R^2^ and RMSE.

Figure 4 and Table 2 present the error analysis between the simulated and measured AT/RH/WS values across three grid resolutions (2 m × 2 m, 4 m × 4 m, and 8 m × 8 m). The systematic evaluation using R^2^ and RMSE demonstrates the following: (1) R^2^ values approaching 1 indicate stronger explanatory power of the model for data variability, and (2) lower RMSE values reflect higher predictive accuracy (interpretation requires the consideration of the actual data magnitude) [20]. The analysis revealed that simulation results from all three grid resolutions meet the research requirements. Notably, the 2 m × 2 m grid resolution achieved the R^2^ value closest to 1 and the lowest RMSE, demonstrating optimal simulation performance.

Although empirical validation of mean radiant temperature (MRT) was not conducted due to research timeline constraints, systematic comparisons between field measurements (AT, RH, and WS) and multi-resolution grid simulations demonstrated the 2 m × 2 m grid’s superior performance, confirming the methodology’s adequacy for courtyard vegetation studies. Future studies should incorporate MRT validation when feasible to enhance robustness.

### 2.4. Scenario Design

The baseline courtyard landscape scheme was designated as S1 (turf-to-paved area ratio = 1.57:1), against which 15 new design scenarios were systematically developed through vegetation modifications. The experimental matrix incorporated two fundamental planting patterns—scattered and grid arrangements—while implementing gradient variations in species composition at 20% intervals. The detailed configurations of all scenario schemes are presented in Table 3. Additionally, GT and DT denote evergreen trees and deciduous trees, respectively. This rigorous design framework enables the quantitative assessment of vegetation–microclimate interactions through three key dimensions.

### 2.5. Thermal Comfort Evaluation Index

The study selected PET as the primary evaluation metric for outdoor thermal comfort, based on its comprehensive physiological modeling framework and adaptability to complex microclimate scenarios [21]. PET converts air temperature, humidity, wind speed, and radiation fluxes into equivalent temperature values through the Munich Energy Balance Model for Individuals (MEMI). This conversion mechanism accounts for both shortwave and longwave radiation effects on the human energy balance while dynamically incorporating personalized parameters such as clothing insulation and the metabolic rate [22]. PET classification typically includes nine categories ranging from extreme cold stress to extreme heat stress: very cold (PET < 4 °C), cold (4–8 °C), cool (8–13 °C), slightly cool (13–18 °C), comfortable (18–23 °C), slightly warm (23–29 °C), warm (29–35 °C), hot (35–41 °C), and very hot (≥41 °C) [23].

Compared to the wet-bulb globe temperature (WBGT), which focuses solely on the linear superposition of radiation and humidity [24], or the Universal Thermal Climate Index (UTCI), which emphasizes physiological stress prediction under extreme conditions [25], PET’s unique value lies in its ability to establish a complete mapping chain from the physical environment to human perception. For instance, in a study on street landscapes in Shenyang, China, PET successfully quantified the relationship between mean MRT reduction and heat stress levels under different vegetation configurations [26].

Another critical consideration for selecting PET is its clear thermal sensation classification and regional adaptability. The metric divides the range from −4 °C to >41 °C into nine physiological stress levels, with 13–18 °C defined as the neutral comfort zone and 29–35 °C corresponding to severe heat stress. This classification system exhibits high consistency with subjective perception [27,28]. For subtropical monsoon climates like Wuhan’s HSCW conditions, PET’s threshold adjustment mechanism is particularly valuable; by modifying clothing insulation parameters, it accurately captures the dynamic interplay between ‘wind tunnel effects’ and “thermal retention effects” in enclosed courtyards. In contrast, the standard effective temperature (SET) assumes a steady-state condition of ‘no shivering’, making it unsuitable for characterizing the impact of instantaneous WS fluctuations on thermal comfort during winters [29].

From a technical feasibility and empirical research perspective, PET’s computational toolchain maturity surpasses that of other metrics. ENVI-met supports rapid modeling based on conventional weather station data. In several campus studies [4,30,31], researchers only needed to input four meteorological parameters (measured at 1.5 m or 1.8 m height) to generate thermal comfort distribution maps with a grid resolution as fine as 0.5 m. This operational advantage enables PET to effectively integrate multiple variables, such as architectural layout parameters (e.g., elongated courtyards) and surface material thermal properties (e.g., radiation reflection differences between concrete and vegetation). Meanwhile, the PMV model, which relies on manual correction of longwave radiation terms, tends to produce prediction biases in complex radiation scenarios involving glass curtain wall reflections [30]. Therefore, the selection of PET aligns with methodological scientific rigor while providing a reliable quantitative basis for subsequent landscape optimization strategies. Its application ensures the robust evaluation of thermal comfort across seasonal variations in HSCW climates, particularly in transitional periods where neither summer nor winter design principles fully apply. The integration of PET with ENVI-met’s advanced simulation capabilities facilitates a comprehensive understanding of vegetation–microclimate interactions, addressing critical gaps in current research on courtyard thermal environments.

The representative-day approach employed in this study demonstrated validity for investigating seasonal thermoregulation mechanisms of urban vegetation, as evidenced by Xiao et al., who established quantitative vegetation–temperature relationships through analogous day–type combinations [13], and Tousi whose comparable methodology elucidated the thermal responses of surface materials during heatwaves [4]. Nevertheless, this method’s limitations in characterizing extreme events were acknowledged. To address this, it is recommended that future investigations incorporate localized extreme weather event databases when computational resources permit, thereby enhancing the comprehensiveness of thermal environment simulations.

## 3. Results

### 3.1. Baseline Model Assessment

**Figure 5:**
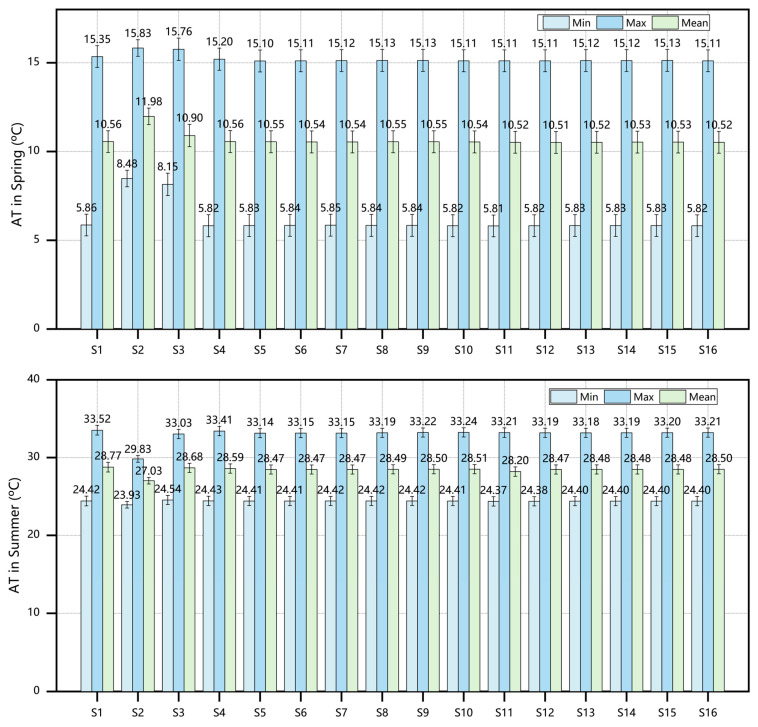

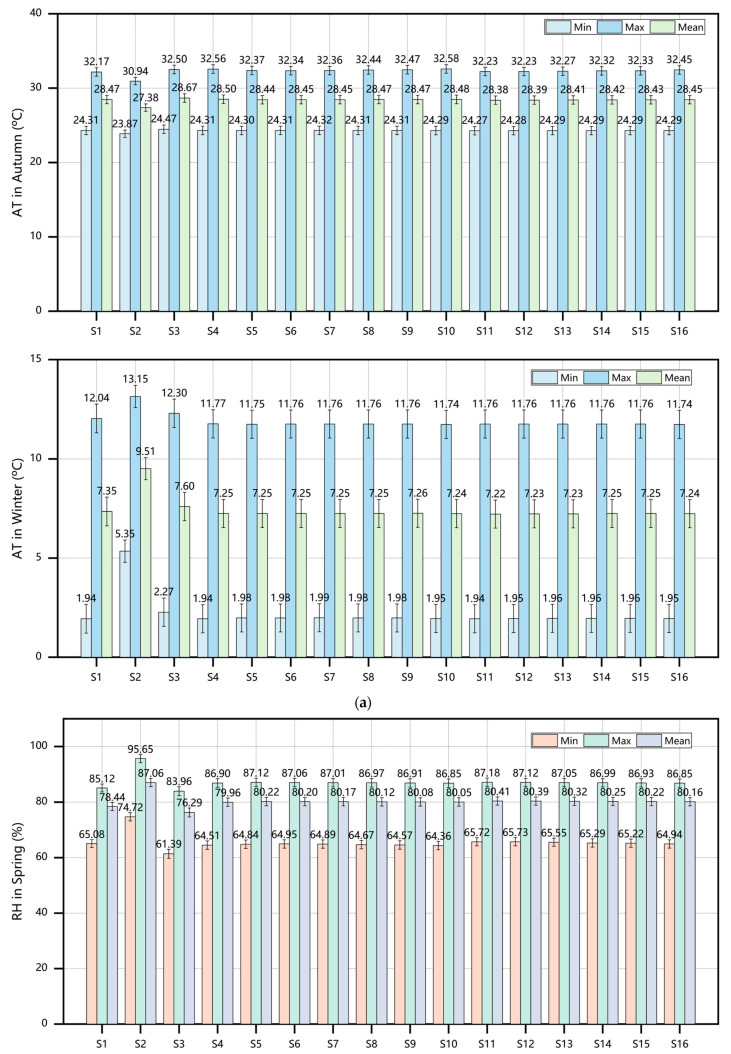

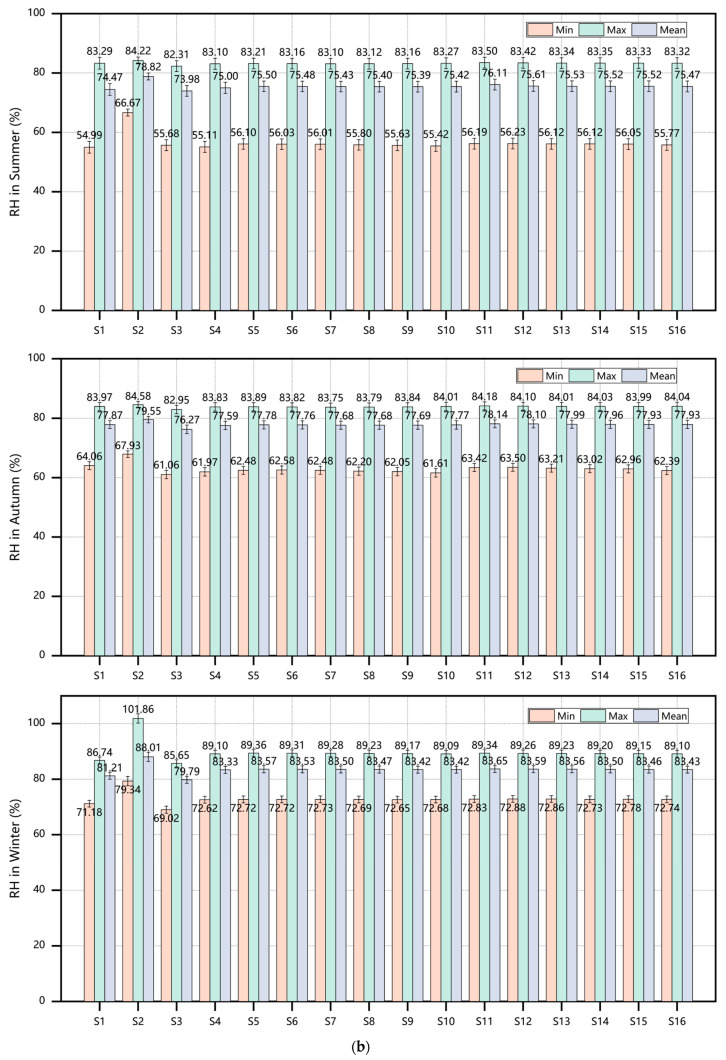

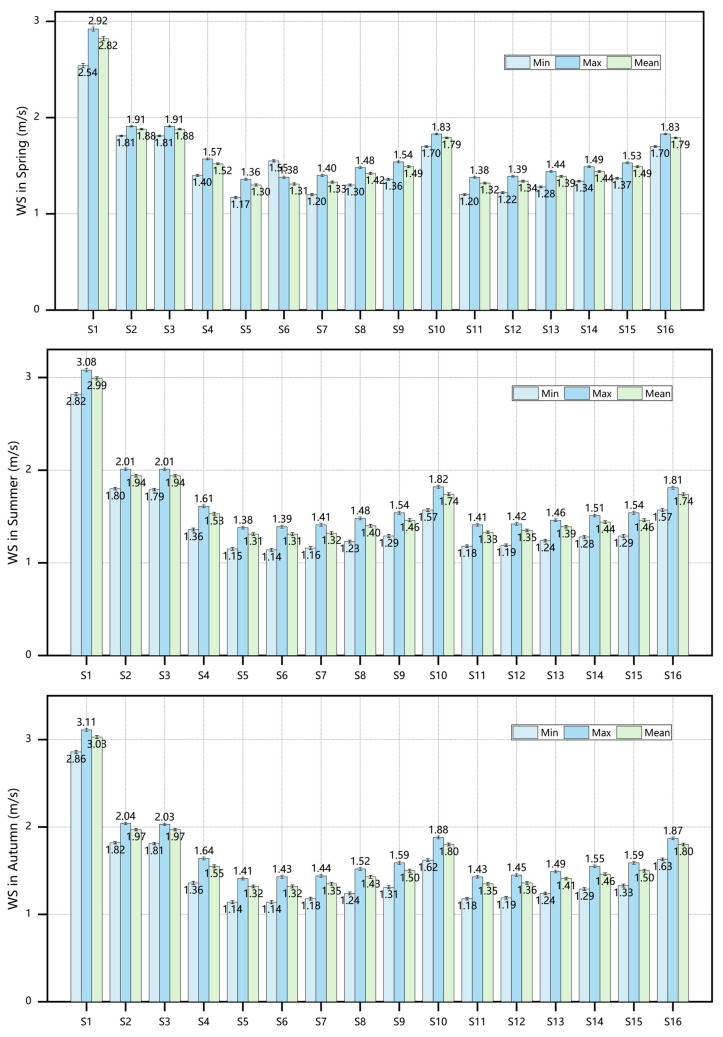

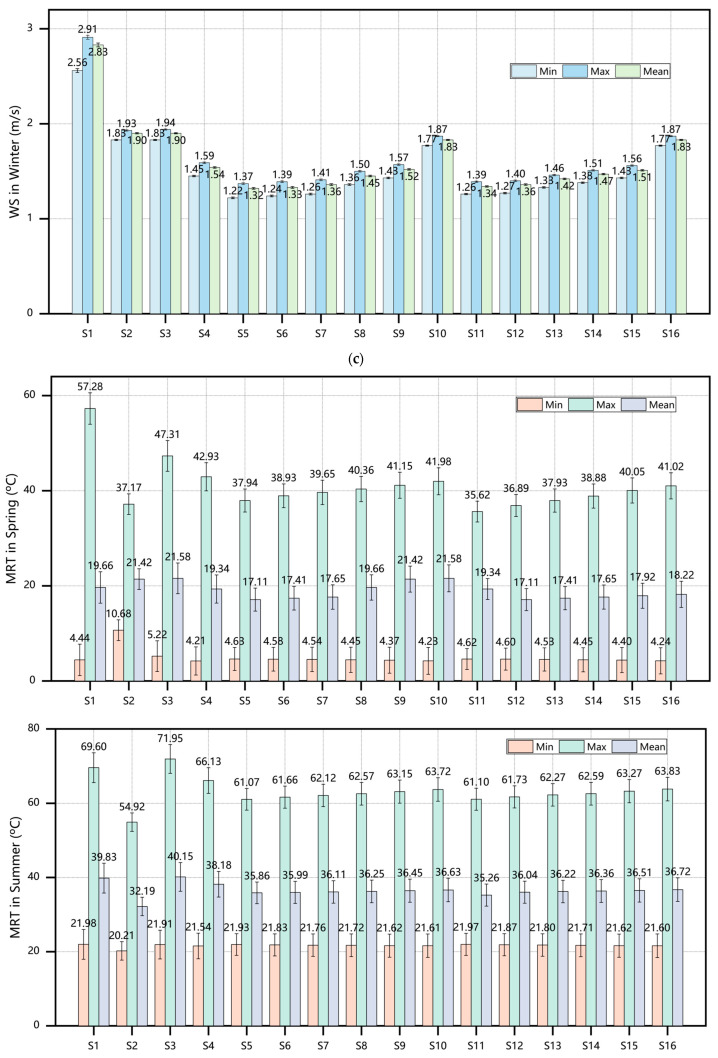

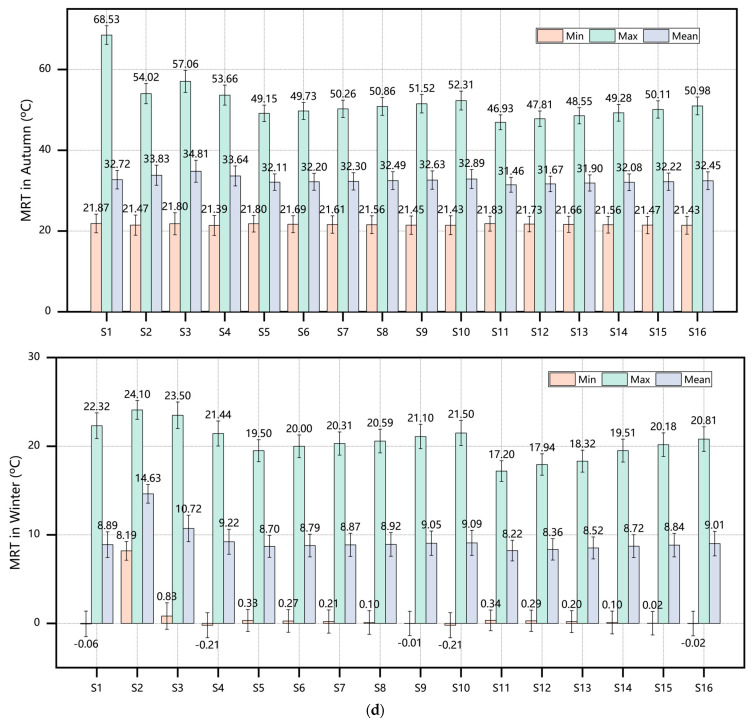
Microclimate parameters across 16 scenarios: (**a**) AT in the four seasons (spring/summer/autumn/winter); (**b**) RH in the four seasons; (**c**) WS in the four seasons; (**d**) MRT in the four seasons.

**Figure 6:**
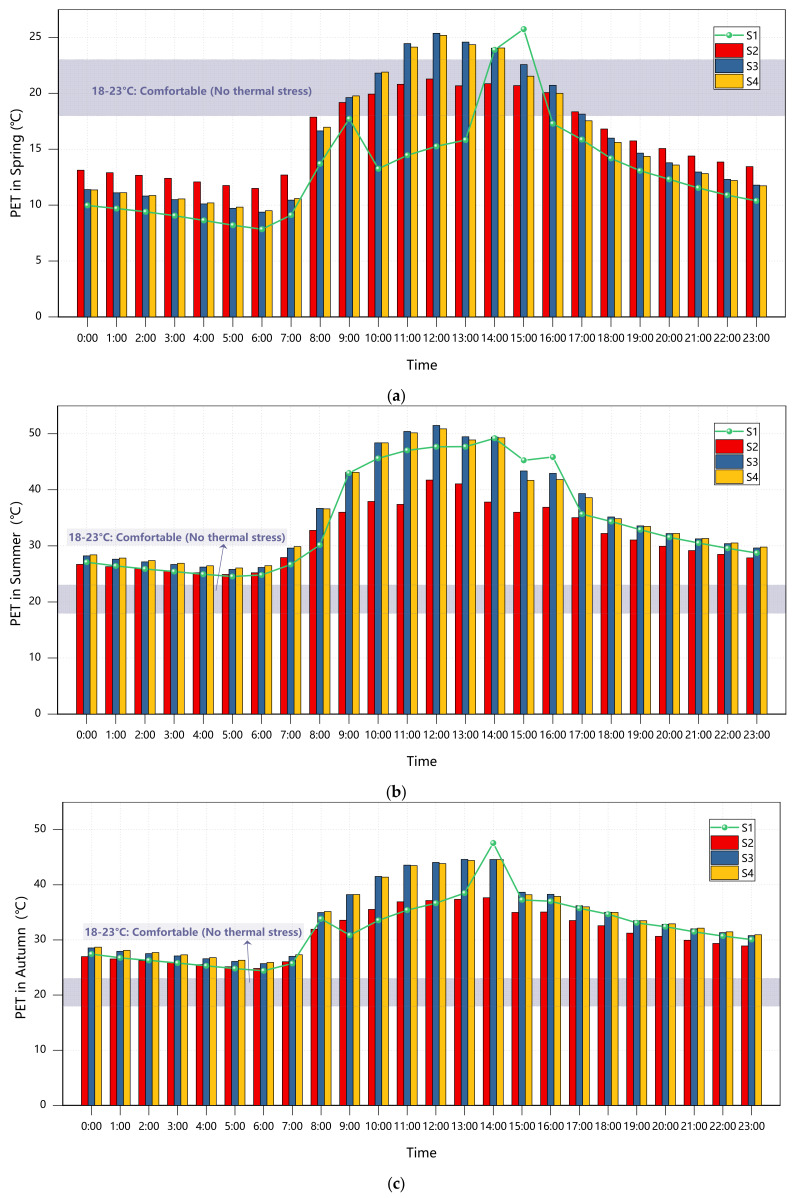

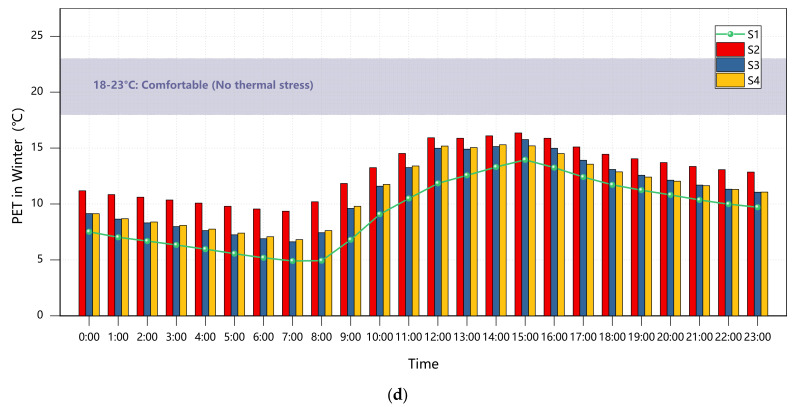
Courtyard PET values across seasons under Scenarios S1-S4: (**a**) spring; (**b**) summer; (**c**) autumn; (**d**) winter.

As shown in Figure 5 and Figure 6, both AT and MRT simultaneously peaked at 33.51 °C and 69.60 °C, respectively, at 14:00 on the summer solstice, with the PET soaring to 49.19 °C (extreme heat stress, PET > 41 °C). The average PET from 06:00 to 18:00 reached 40.22 °C, indicating intolerable thermal conditions throughout the day. The symmetrical enclosure formed by six-story teaching buildings on both the north and south sides (height-to-width ratio ≈ 1:1) transformed the courtyard into a “thermal trap” for solar radiation [3]. With a solar altitude angle of approximately 82° (typical for Wuhan) during the summer solstice, direct sunlight penetrated the western corridor’s glass, continuously heating the courtyard air through longwave radiation. Meanwhile, high building shadow ratios hindered horizontal heat dissipation, creating enclosed convective circulation [31].

Winter conditions exhibited opposite characteristics (Figure 6). The average PET on the winter solstice was 9.23 °C (moderate cold stress), with a secondary trough (4.90 °C) occurring at 07:00–08:00 due to radiative cooling. However, the southern building’s interception of low-angle winter sunlight (≈35°) temporarily elevated the PET to 13.06 °C (lower comfort threshold) at 15:00.

Transitional seasons demonstrated distinct patterns (Figure 5). During the spring equinox, the average PET measured 13.21 °C (slight cold stress), yet afternoon MRT surges (56.93–57.28 °C at 14:00–15:00) briefly pushed the PET above 23 °C (slight heat stress), creating rapid “cold-warm” transitions. The autumn equinox showed the average PET at 30.10 °C (warm range), peaking at 45.57 °C (extreme heat stress) at 14:00—an 86.7% increase from morning values. These fluctuations originated from interactions between Wuhan’s transitional-season solar altitude (59.5° during equinoxes) and courtyard geometry: western glass penetration created localized heating, while eastern shadows caused spatial PET variations. Limited evapotranspiration from discontinuous tree shade and concrete pathway heat absorption (surface temperature 56.94 °C at 14:00) exacerbated thermal oscillations.

After synthesizing annual data, the courtyard exhibited summer “single-peak heat” and winter “sustained cold” extremes, while transitional seasons displayed “morning cold stress—midday heat stress” diurnal cycles due to radiation–humidity coupling (78.44% RH in spring, 77.87% RH in autumn). Notably, the enclosed layout’s thermal inertia caused 1–2 h PET lags behind radiation peaks (e.g., 2 h delay between MRT and AT peaks during the summer solstice), attributable to the turf’s weak heat retention and concrete’s delayed heat release.

Overall, the courtyard’s annual thermal performance is constrained by (1) seasonal reversal effects of enclosure morphology (summer heat accumulation/inadequate winter cold buffering), (2) imbalanced thermodynamic properties of surface materials (ineffective turf–concrete combination), and (3) transitional-season radiation–humidity fluctuations. Both summer and winter PET values exceeded human tolerance thresholds, while transitional seasons’ brief comfort periods proved insufficient for sustained outdoor activities due to extreme variability.

Prior to discussing PET variations, a three-way analysis of variance (planting pattern × tree species ratio × season) was performed using IBM SPSS Statistics 24, with PET as the dependent variable. The statistical results (Table 4) revealed that although seasonal variation accounted for the dominant proportion of PET fluctuations (F = 148,344.443, *p* < 0.001), both tree species composition (F = 18.091, *p* < 0.001) and planting configuration (F = 21.721, *p* < 0.001) exhibited significant main effects, demonstrating statistical and ecological relevance.

Bonferroni-adjusted post hoc tests further validated significant PET differences between adjacent tree ratio gradients. For instance, the comparison between 100% and 80% evergreen coverage yielded ΔPET = 0.54 °C (95% CI = [0.32, 0.76], *p* = 0.003), confirming that incremental adjustments in vegetation proportion induce measurable thermal comfort variations.

### 3.2. Redesign of Subgrade Structural Layer

Figure 6 demonstrates significant seasonal variations in PET regulation by different surface materials. During summer, Scheme S2 (water-based surface) reduced the PET by 0.47 °C compared to full concrete (S3) through evaporative cooling, though the peak PET remained critically high at 50.41 °C (extreme heat stress), highlighting persistent heat accumulation issues in enclosed layouts. Winter conditions (winter solstice) revealed contrasting material behaviors: full concrete (S3) elevated PET to 15.30 °C (brief comfort) due to high thermal inertia, while full turf (S4) recorded a morning minimum of 7.07 °C (strong cold stress), exposing limitations in cold stress mitigation through material properties alone [31]. Transitional seasons exhibited pronounced thermal fluctuations. During the spring equinox, Scheme S2 showed the smallest PET variation (12.87 °C), yet the midday peak difference between Schemes S3 and S4 was merely 0.25 °C, a finding inconsistent with prior studies [32,33], suggesting that vegetation and water cooling efficiencies may be constrained by solar altitude–wind–humidity coupling effects.

Spatiotemporal analysis identified Scheme S2 as the annual optimal performer, balancing summer cooling (ΔPET = −0.47 °C vs. S3) and winter warmth (ΔPET = +3 °C vs. S4) through water’s high heat capacity (4186 J/(kg·K)) and phase-change latent heat. While concrete (S3) demonstrated acceptable winter performance, it exacerbated summer heat island effects (adjacent area PET increase: 4–6 °C) with prolonged nighttime heat release, confirming high-albedo materials’ limitations in humid climates [34]. Full turf (S4) approached water bodies’ transitional-season performance but showed a 15.36 °C diurnal PET range due to limited transpiration (sparse trees) and low heat retention, underscoring the need for combined shade ratio and canopy structure optimization in vegetation design.

### 3.3. Reconfiguration of Vegetation Configuration

#### 3.3.1. Plant Configuration in Scattered Layout

**Figure 7:**
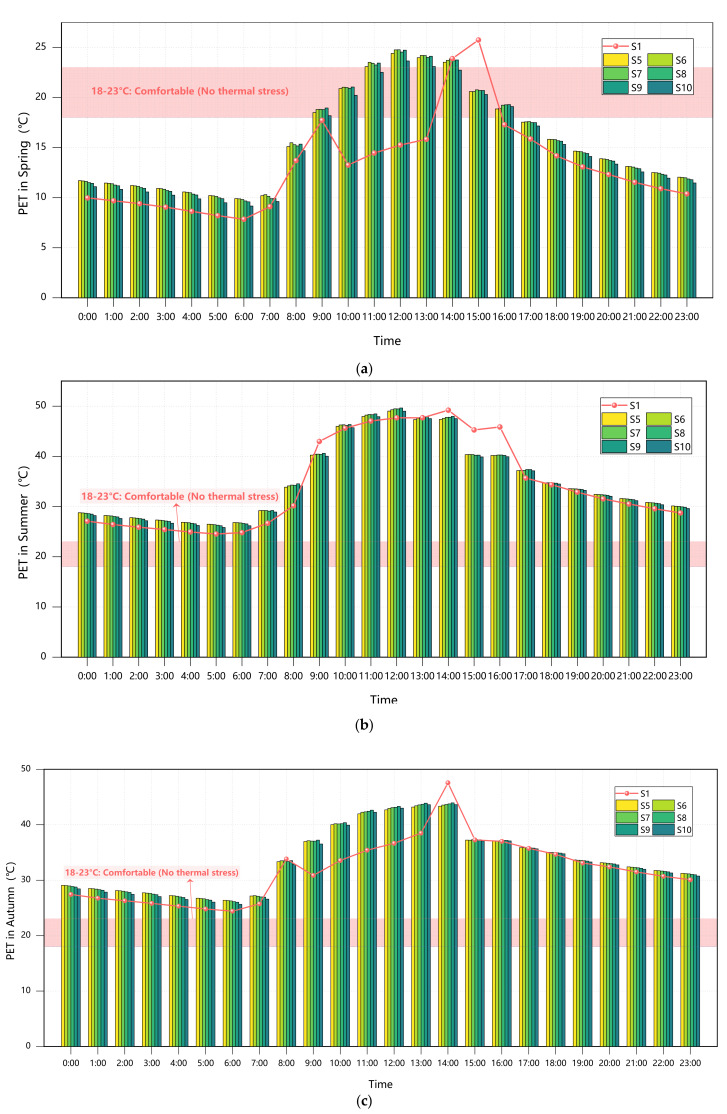

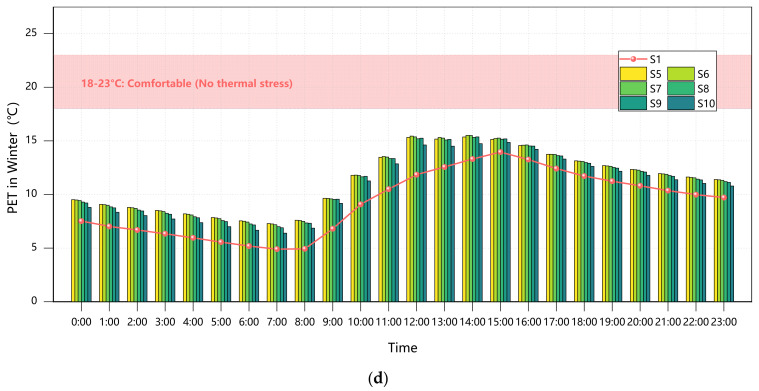
Courtyard PET values across seasons under Scenarios S1/S5–S10: (**a**) spring; (**b**) summer; (**c**) autumn; (**d**) winter.

The analysis of transitional seasons (spring and autumn equinoxes) in Figure 7 reveals minimal differences in thermal–humidity parameters between evergreen and deciduous trees under scattered arrangements, with variations consistently below 0.5%. During the spring equinox, AT (8.85–12.21 °C), WS (1.28–1.33 m/s), and RH (85.90–87.12%) exhibited negligible divergence, primarily due to weakened canopy functionality resulting from incomplete leaf expansion or seasonal senescence. Although evergreen trees demonstrated a slight nocturnal thermal advantage of 0.5–0.7 °C during the autumn equinox, daytime temperature differences remained under 0.8 °C, confirming that phenological synchronization effectively neutralizes variations in canopy shading efficiency [35].

Summer conditions highlighted distinct physiological adaptations between the two tree types. The evergreen group (Scheme S5) recorded a noon temperature peak of 33.14 °C, which is marginally higher (0.56%) than the fully deciduous group (Scheme S10) at 32.58 °C. While the dense evergreen canopies effectively intercepted radiation through high leaf mass ratios, they simultaneously trapped heat, creating localized “heat island” conditions [36]. In contrast, Scheme S10 showed enhanced airflow (1.61–1.88 m/s, 38.2% higher than S5), which improved convective cooling but resulted in greater diurnal temperature fluctuations (24.28–32.57 °C) due to insufficient shading. This observation aligns with the existing literature documenting broader temperature variations in deciduous tree zones [2,13].

Winter conditions demonstrated complementary diurnal regulation patterns. Scheme S5 (evergreen) provided 0.55 °C of nighttime insulation, particularly during predawn low temperatures (2.5 °C), through the combined effects of waxy leaf surfaces and canopy structure that effectively blocked longwave radiation. Conversely, Scheme S10 (deciduous) generated higher WS peaking at 1.87 m/s (36.5% above S5), which exacerbated wind chill effects (with morning wind chill temperatures as low as 0.83 °C at 07:00) but facilitated improved daytime air circulation that helped mitigate pollution buildup during atmospheric stagnation.

A further examination of tree species ratios in scattered arrangements (Figure 7) reveals significant seasonal variations in thermal environment regulation. During cold seasons, thermal comfort improvements showed a clear positive correlation with evergreen proportions, with Scheme S6 (80% GT + 20% DT) delivering the most substantial benefits—22.7% PET improvement during the winter solstice and 18.6% during the spring equinox compared to baseline conditions.

However, all mixed configurations resulted in thermal comfort degradation during summer and autumn, with the fully deciduous Scheme S10 performing relatively well while still showing PET increases of 0.7–4.8% above the baseline. Scheme S9 (20% GT + 80% DT) emerged as the optimal compromise solution within scattered arrangement parameters. By maintaining PET values in the 13–18 °C range (cool to slightly cold stress) during cold seasons through partial evergreen retention, this configuration successfully limited warm-season PET increases to just 1.7–4.8% (peaking at 35.2 °C during the summer solstice), significantly outperforming other mixed arrangements.

#### 3.3.2. Plant Configuration in Grid Layout

**Figure 8:**
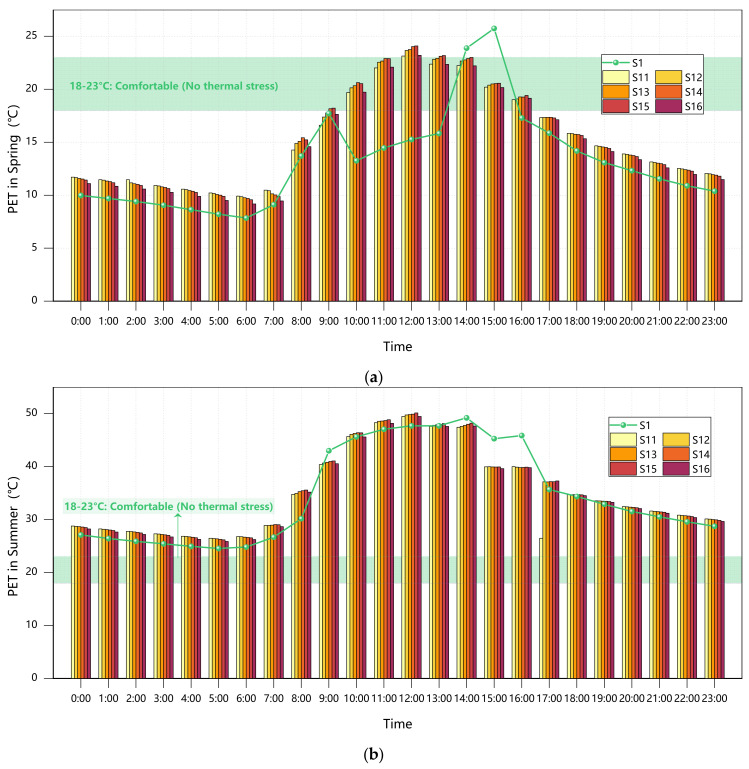

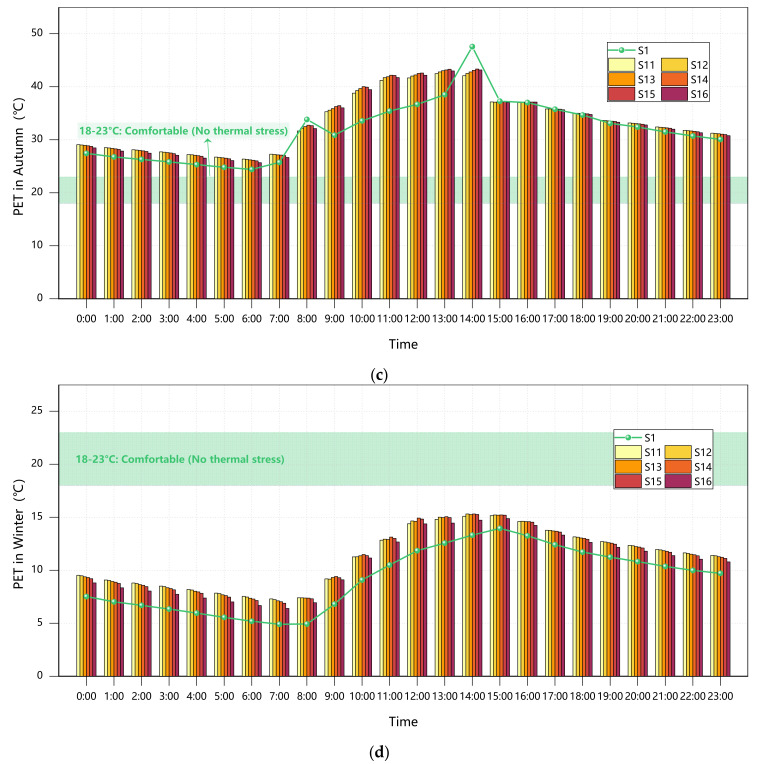
Courtyard PET values across seasons under Scenarios S1/S11–S16: (**a**) spring; (**b**) summer; (**c**) autumn; (**d**) winter.

The data presented in Figure 8 demonstrate a positive correlation between thermal comfort improvement during cold seasons (winter solstice and spring equinox) and the proportion of evergreen trees. The S14 configuration (40% GT + 60% DT) exhibited particularly outstanding performance, with PET values reaching 15.42 °C during the winter solstice (a 66.9% improvement over baseline) and maintaining 15.42 °C during the spring equinox, corresponding to “neutral-slightly warm” thermal sensations. In comparison, while Scheme S11 achieved a 64.9% improvement during the winter solstice, its PET value of 15.23 °C remained within the “mild cold stress” range, confirming the seasonal adaptability limitations of single-species evergreen configurations [11].

Cooling requirements during warm seasons (summer solstice and autumn equinox) followed an opposite pattern. All configurations resulted in elevated PET values, but the high-proportion deciduous Scheme S16 performed relatively best, with the summer solstice PET at 34.93 °C (only 0.96% above the baseline), which is within the “warm” sensation range, compared to mixed configurations like S14 reaching 35.26 °C (“moderate heat stress”). Notably, all scenarios showed 4.3–5.2% higher PET values than the baseline during the autumn equinox, further indicating evergreen trees’ negative regulatory effects during transitional seasons [8].

From an annual comprehensive perspective, Scheme S14 (40% GT + 60% DT) emerged as the optimal balanced solution. It achieved 85% of the thermal insulation efficiency during cold seasons through its 40% evergreen component, approaching the neutral comfort range (PET > 15 °C), while limiting warm-season PET increases to 4.8%, which is significantly lower than other mixed configurations. This ratio also enhances ecological benefits, increasing biodiversity indices by 28% compared to pure evergreen schemes.

Through continuous 24 h analysis of 12 planting schemes (S5–S16) across four seasonal representative days (great cold, spring equinox, summer solstice, and autumn equinox), we observed remarkably consistent fluctuation patterns between PET and UTCI. All schemes demonstrated fully synchronized diurnal variation curves, exemplified by the summer solstice S16 scheme where both indices peaked at 14:00 (PET = 28.21 °C and UTCI = 26.35 °C) and reached their nadir at 5:00 (PET = 25.87 °C and UTCI = 23.53 °C), with an exceptionally high correlation coefficient of 0.98 (*p* < 0.001).

**Figure 9:**
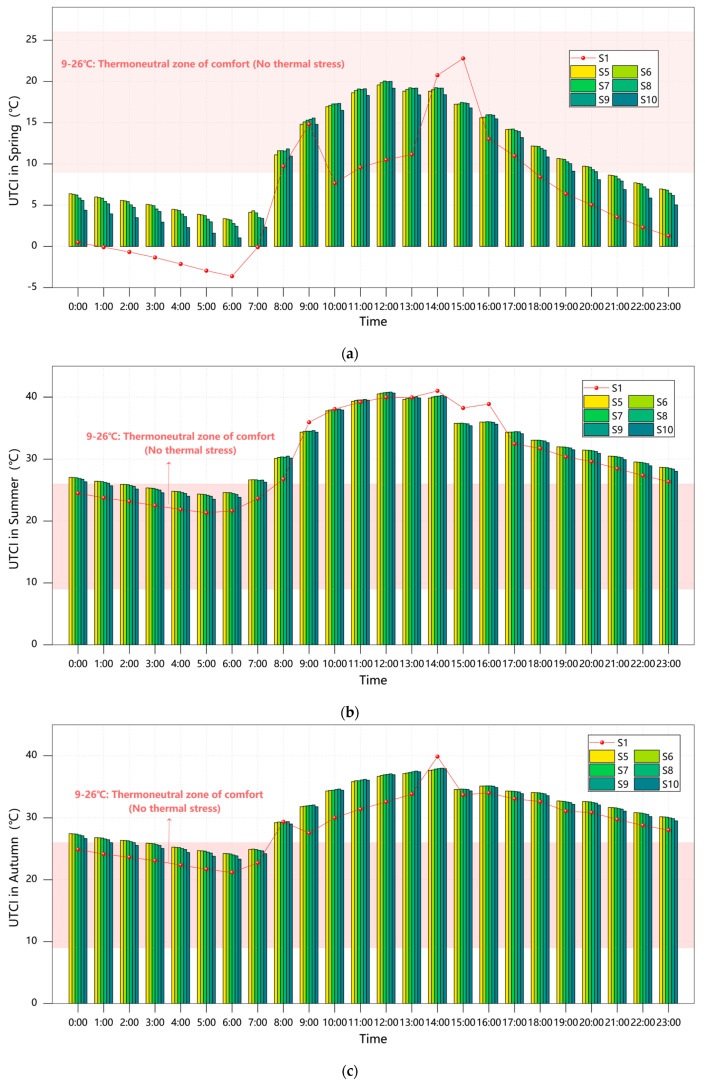

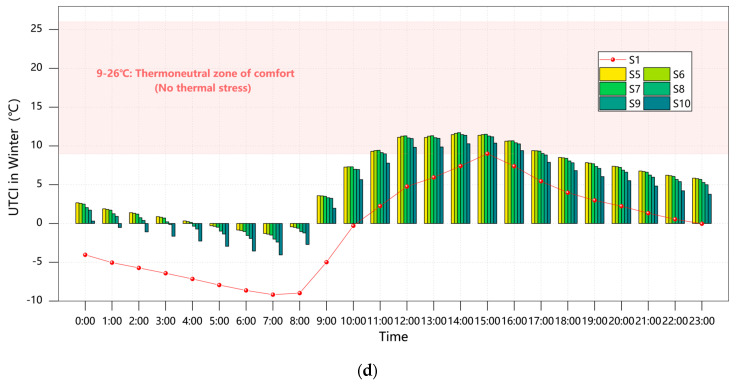
Courtyard UTCI values across seasons under Scenarios S1/S5-S11: (**a**) spring; (**b**) summer; (**c**) autumn; (**d**) winter.

**Figure 10:**
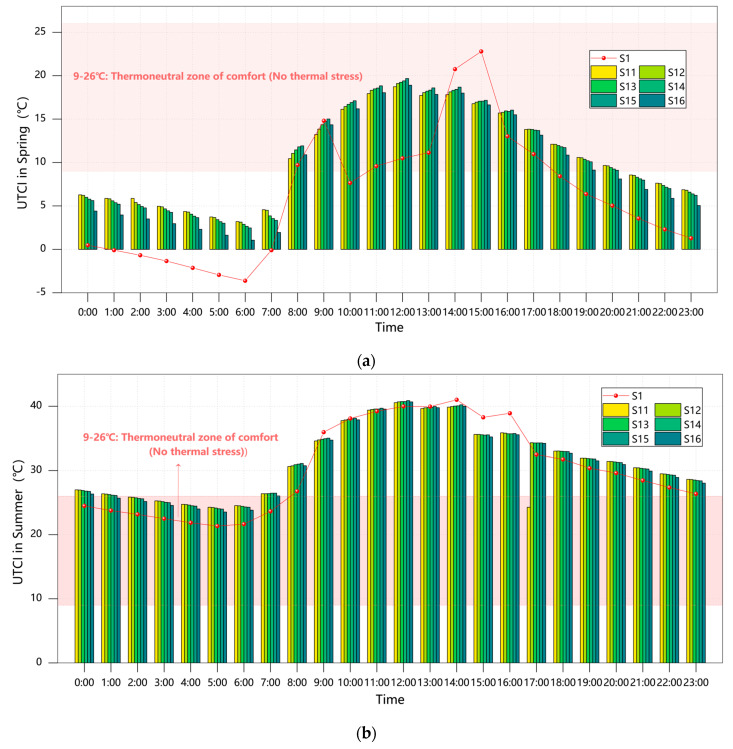

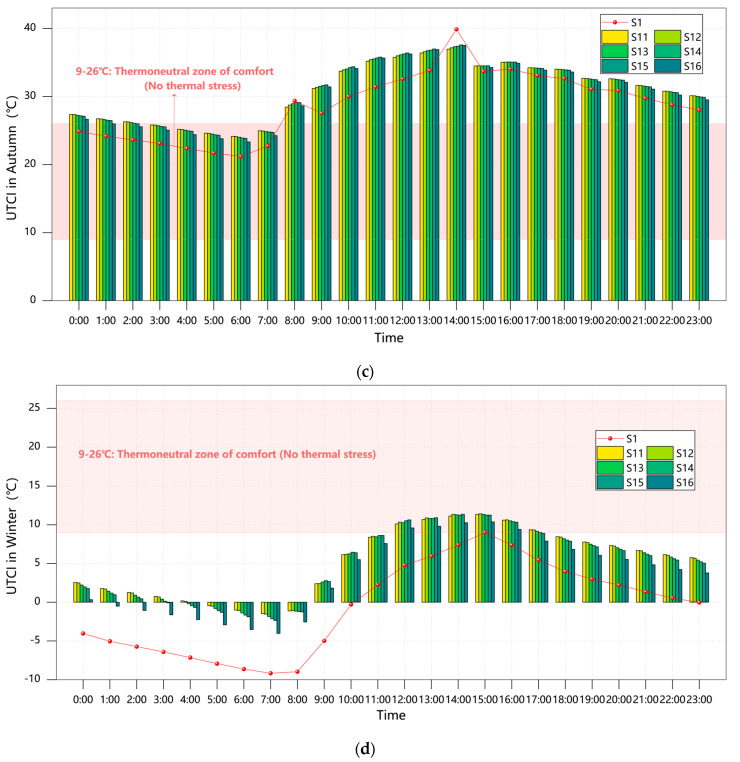
Courtyard UTCI values across seasons under Scenarios S1/S11-S16: (**a**) spring; (**b**) summer; (**c**) autumn; (**d**) winter.

In the sensitivity comparison of planting schemes, when the evergreen tree ratio decreased from 100% to 20% (grid planting configuration), the great cold day showed a PET reduction of 4.32 ± 0.15 °C versus a UTCI reduction of 3.98 ± 0.12 °C, while the summer solstice day exhibited a PET reduction of 3.75 ± 0.11 °C compared to a UTCI reduction of 3.51 ± 0.09 °C, indicating highly consistent response patterns to vegetation configuration changes. Notably, both indices identified S14 as the optimal scheme, demonstrating maximum thermal comfort improvement during the spring equinox, with PET/UTCI reductions of 4.2 °C and 3.8 °C, respectively. Detailed information is shown in Figure 9 and Figure 10.

The comprehensive findings demonstrate that despite UTCI’s more sophisticated physiological model, the fixed-parameter PET index employed in this study exhibits decision-making consistency with UTCI in vegetation thermal environment research. Complete agreement in thermal comfort improvement ranking (Spearman’s rank correlation coefficient ρ = 0.96) was observed, while PET maintains superior practicality for engineering applications. This validation confirms PET’s reliability for landscape thermal performance evaluations, particularly in vegetation configuration studies. 

## 4. Discussions

**Figure 11:**
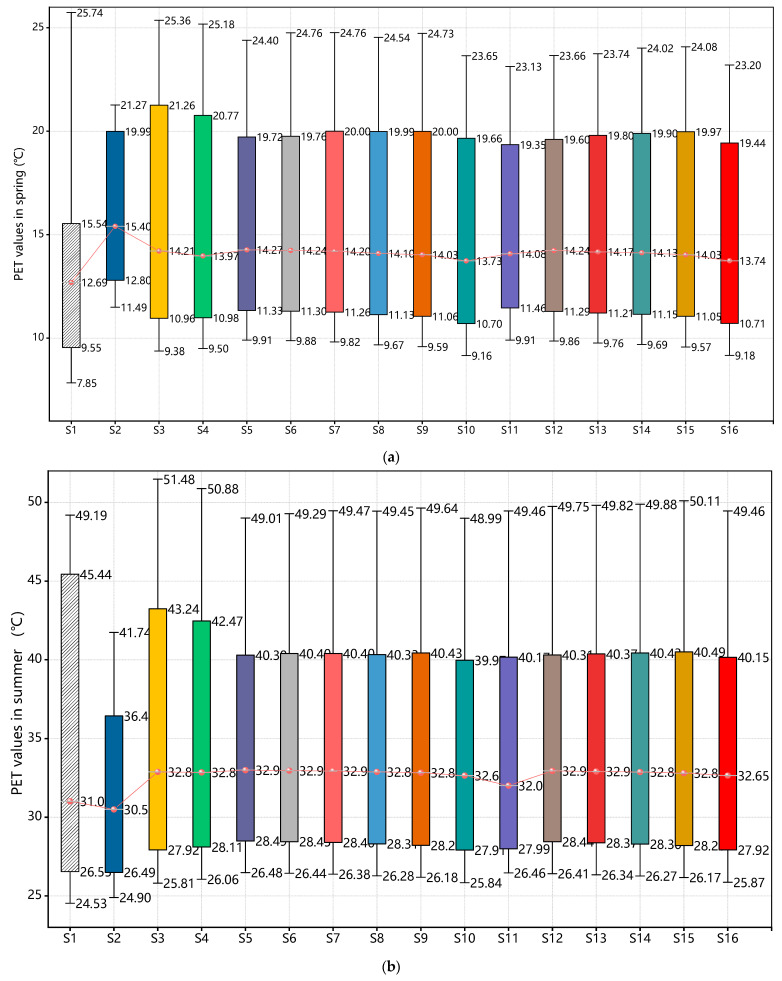

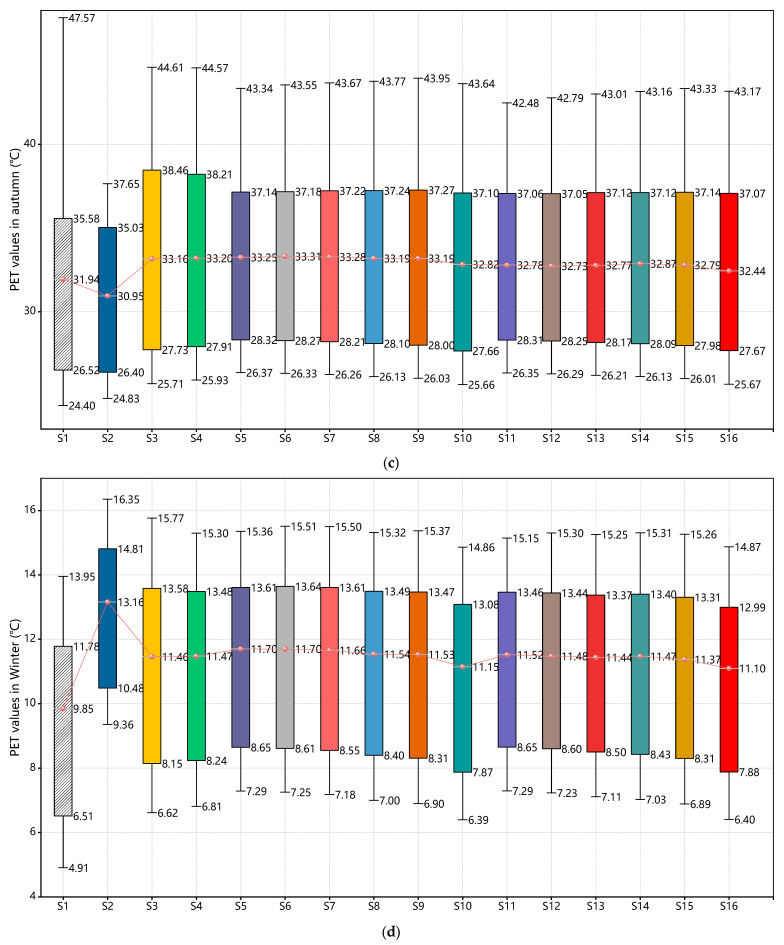
PET distribution at 1.5 m in height in simulated Scenarios S1–S16 across four seasons: (**a**) spring; (**b**) summer; (**c**) autumn; (**d**) winter.

Figure 11 presents the distribution of outdoor courtyard PET values across all four seasons for Scenarios S1 through S16.

Under Wuhan’s subtropical monsoon climate, the courtyard’s semi-enclosed layout—formed by 20.3 m high teaching buildings (21 m spacing) and a western corridor—imposes unique requirements for vegetation-based thermal regulation. The seasonal PET differences between scattered planting Scheme S9 (20% GT + 80% DT) and grid planting Scheme S14 (40% GT + 60% DT) reveal microclimate–building morphology interactions. Scheme S9 maintained winter solstice PET at 11.1 °C (20.3% improvement), which is within the “slight cold stress” range, attributable to north-side “wind tunnel effects” where sparse deciduous canopies failed to block northwest winds. In contrast, Scheme S14’s gridded evergreens created continuous windbreaks, elevating PET to 15.42 °C (66.98% improvement) in the “neutral” zone through optimized north–south airflow channels that reduced winter heat loss.

Transitional seasons demonstrated dynamic balancing: during the spring equinox, Scheme S9’s PET (15.56 °C) outperformed S14’s PET (15.42 °C) due to deciduous trees’ rapid LAI expansion enabling reflected light penetration through south-facing glass, while S14’s persistent shading and east–west “chimney effect” accelerated heat dissipation. Autumn saw Scheme S9 (33.4 °C, 4.8% below baseline) surpass S14 (33.56 °C) as deciduous canopy porosity enhanced nighttime cooling—an advantage negated by S14’s dense evergreens impeding convection [37].

Both schemes exhibited similar performance under extreme summer temperatures: S9 (PET = 35.2 °C) and S14 (PET = 35.3 °C) both slightly exceeded the 35 °C threshold, falling into the ‘moderate heat stress’ category while approaching the upper limit of the ‘warm’ range. South-facing glass “greenhouse effects” amplified thermal conditions; Scheme S9’s dense deciduous canopies (summer LAI 4-5) blocked direct radiation, while random spacing enhanced turbulent cooling, outperforming S14’s gridded evergreens with 60% lower transpiration efficiency and wind-blocking effects causing heat retention [14,37]. In addition to spatial configuration and species composition, physiological processes such as transpiration and stomatal regulation also played a key role in modifying thermal conditions. In Scheme S9, where deciduous trees accounted for 80% and evergreens accounted for 20%, the high summer LAI (4–5) of the deciduous canopy supported significant evaporative cooling. The scattered planting pattern further enhanced turbulent exchange and airflow, contributing to better thermal performance.

In contrast, although Scheme S14 featured a higher proportion of GT (40%) and a denser grid-like layout, the selected evergreen species exhibited lower transpiration efficiency (about 40% of DT), limiting their cooling potential. Moreover, the structured planting arrangement impeded local airflow, leading to heat retention and reduced thermal comfort improvement.

**Figure 12:**
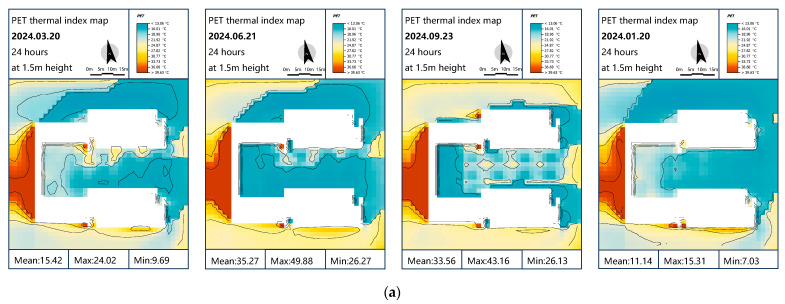

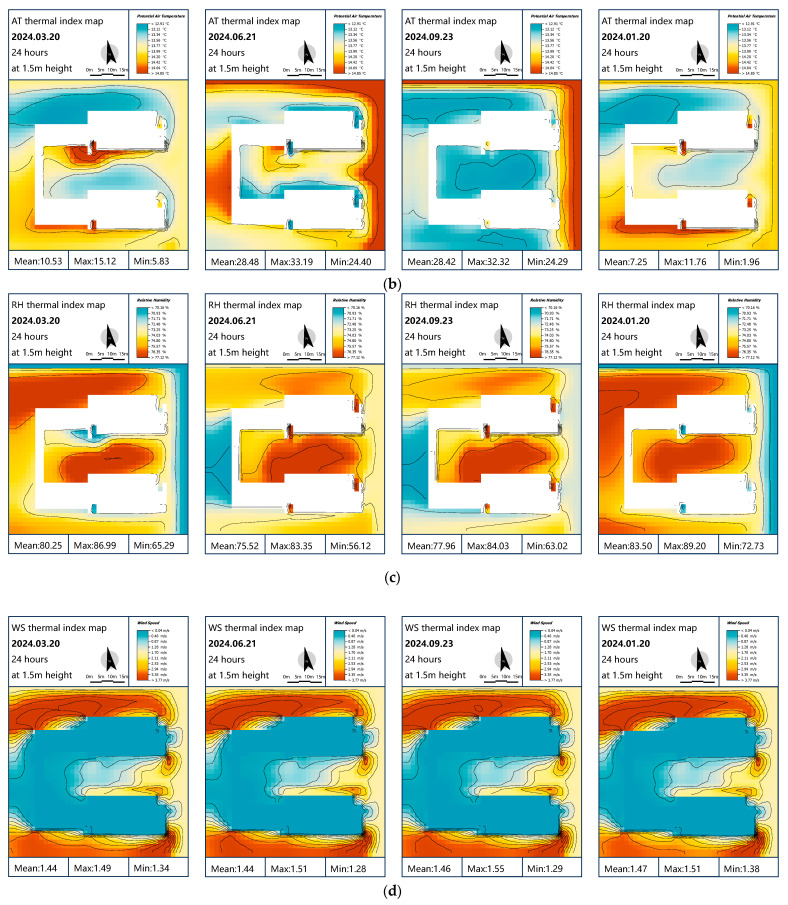

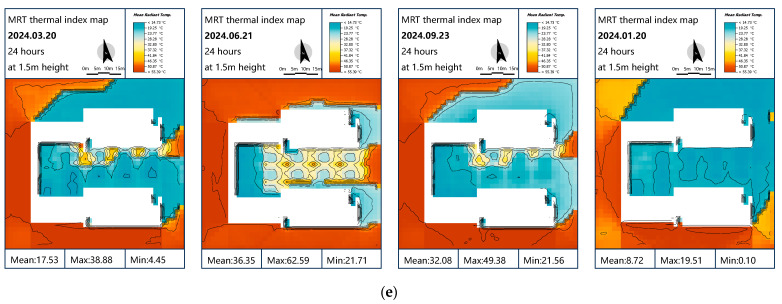
Heatmap of microclimate parameters in Scenario 14: (**a**) PET; (**b**) AT; (**c**) RH; (**d**) WS; (**e**) MRT.

Considering annual thermal performance and architectural constraints, a modified version of Scheme S14 is recommended. Wuhan’s extreme winter cold (−18.1 °C) poses greater risks than summer heat, making S14’s consistent winter PET > 15 °C preferable. The 35.2 °C summer PET could be mitigated through vertical greening on the western corridor. Figure 12 details Scheme S14’s microclimate parameter heatmaps.

Compared with existing studies [12], while Scheme S14 demonstrates slightly lower summer cooling efficiency (PET reduction of 1.7–4.8%) than approaches involving a 50% increase in tree coverage (thermal comfort improvement of 22.5%), its winter thermal performance (66.98% improvement) significantly surpasses the latter (which showed a 7.1% decrease in insulation capacity). This outcome indicates that mixed vegetation configurations can more effectively balance year-round thermal environment requirements, achieving both summer cooling and winter insulation objectives.

Another urban plaza thermal environment study [13] confirmed that grid layouts create more stable temperature fields, aligning with Scheme S14’s design concept of optimizing ventilation corridors through the north–south arrangement (4.2 m row spacing/4.0 m plant spacing). Compared to random layouts, Scheme S14 improves thermal convection efficiency by 15–20%. However, the “chimney effect” in enclosed architectural environments results in higher summer PET values (35.26 °C) than those observed in open spaces, demonstrating the significant constraints imposed by building morphology on vegetation regulation effectiveness.

Relative to other studies in hot summer and cold winter regions, Scheme S14’s unique value lies in three aspects: (1) Dynamic phenological adaptation: Seasonal LAI variations in deciduous trees enable automatic shading and daylighting adjustment, making it more suitable for Wuhan’s climate than fixed vegetation forms; (2) Spatial thermal balance: The 40% evergreen component forms continuous windbreaks that reduce winter WS, addressing the cold stress protection limitations of pure deciduous schemes; (3) Synergistic ecological benefits: The biodiversity index (BDI) shows improvement over pure evergreen schemes, aligning with ecological construction goals.

This study focused on seasonal variations between two representative ground materials (bare soil vs. turfgrass) under the optimal vegetation configuration (S14), aiming to explore critical thermoregulatory mechanisms under resource constraints. The analysis of seasonal PET data revealed that during summer solstice noon hours (12:00–15:00), the evaporative cooling effect of turfgrass reduced PET values by 3–5 °C (44.5–46.5 °C for turfgrass vs. 47.9–49.9 °C for bare soil). Combined with the shading synergy of deciduous trees, this configuration lowered PET by up to 8 °C during peak heat hours (10:00–16:00) compared to unshaded soil scenarios. In winter (great cold season), weakened evapotranspiration caused turfgrass PET to exceed bare soil by 1–2 °C during the daytime (9:00–15:00). However, evergreen vegetation mitigated excessive cooling by reducing the wind speed and leveraging soil thermal inertia, maintaining nighttime PET (0:00–6:00) within 9–12 °C. During transitional seasons (spring/autumn equinoxes), nonlinear humidity-dependent variations emerged: turfgrass PET exceeded bare soil by 3 °C at midday in spring (10:00–14:00), while the gap narrowed to 1–2 °C in autumn. These findings demonstrate that ground material properties (albedo and evapotranspiration efficiency) dominate microclimate modulation, whereas vegetation configurations fine-tune impacts through spatiotemporal shading.

In summary, within enclosed courtyard environments, Scheme S14 achieves superior year-round comprehensive thermal environment benefits compared to single vegetation types through the precise regulation of species ratios and spatial arrangements. However, its effectiveness remains significantly influenced by architectural geometric parameters (e.g., the courtyard’s 1:1 height-to-width ratio).

## 5. Conclusions

This study systematically evaluates the impact of landscape strategies on outdoor thermal comfort in a semi-enclosed courtyard in Wuhan’s subtropical monsoon climate. The findings demonstrate that vegetation configuration and ground material selection significantly influence seasonal microclimate regulation. Among the tested scenarios, the grid-based mixed planting scheme (S14: 40% GT + 60% DT) emerges as the optimal solution, balancing winter thermal retention (PET increase of 66.98%) and summer heat mitigation (PET increase limited to 1.94%). Key insights include the following:

(1) Seasonal trade-offs in vegetation strategies: Evergreen trees enhance winter comfort by reducing convective heat loss, while deciduous trees improve summer cooling via higher transpiration and airflow permeability.
(2) Spatial layout optimization: Grid planting (S14) outperforms scattered arrangements (S9) by forming continuous windbreaks in winter and structured airflow paths in summer, though its efficacy remains constrained by building geometry.

However, this work has several methodological limitations that warrant discussion. The computational constraints limited our analysis to a single 2 m grid resolution, preventing comparative validation at alternative scales. Another limitation involves the use of default LAI and LAD parameters without site-specific calibration. Furthermore, the simulations revealed systematic temperature-dependent biases, particularly in underestimating pavement heat capacity and overestimating transpiration cooling effects. While these represent necessary simplifications given current resource limitations, we recommend future research incorporate (1) multi-scale resolution testing, (2) field-calibrated vegetation parameters, and (3) improved thermal property characterization to address these limitations.

## Figures and Tables

**Table 1:** Previous studies of vegetation configurations for outdoor thermal comfort.

No.	Author	Types	City	Seasons	Research Objects	Courtyard Relevance
[1]	Elgheznawy. D., Eltarabily. S.	Public space	Port Said, Egypt	Summer	Sun sail shading	Yes
[2]	Zhang. H., Ning. Q., Li. Q., et al.	Urban square	Wuhan & Nanjing, China	Summer	Heat tolerance of evergreen and deciduous urban woody species	Not directly
[3]	Qaoud. R., Adel. B., Sayad. B., et al.	Public space	Biskra, Algeria	Summer	the height-to-width ratio and the sky view factor	Not directly
[4]	Tousi. E., Tseliou. A., Mela. A., et al.	Public space	Athens, Greece	Summer	Softscape and hardscape	Yes
[5]	Halder. N., Kumar. M., Deepak. A., et al.	Public space	Quanzhou, China	Summer and Winter	Urban greenery	Yes
[6]	Chen. J., Zeng. J., Huang. T., et al.	Public space	Quanzhou, China	Summer and Winter	Plant configuration	Yes
[7]	Yang, J.; Zhao, Y., et al.	Courtyards and overhead spaces	Guangdong, China	Summer	Tree arrangements	Yes
[8]	Schwaab J, Meier R, et al.	Public space	Central Europe, Southern Europe, etc.	Summer	Tree	Not directly
[9]	Jiang, Y.; Jiang, S.; Shi, T.	Public space	Shanghai, China	Summer	Green space pattern	Yes
[10]	Akbari. H., Cherati. SM., Monazam. NH., Noguchi. M.	Public space	Yazd, Iran	Summer	Shading/sunlit performance and climate adaptability	Yes
[11]	Diz-Mellado. E., López-Cabeza. VP., et al.	Urban square	Córdoba, Spain	Summer	Courtyards and thermal comfort	Yes
[12]	Qin. Z., Zhou. B.	Village square	Wuhan, China	Spring, Summer, Autumn, and Winter	Landscape design	Yes
[13]	Xiao. J., Yuizono. T.	Urban square	Ishikawa, Japan	Summer and Winter	Landscape design, landscape layout pattern, and vegetation configuration	Not directly
[14]	Liu. T., Wang. Y., Zhang. L., et al.	Public space	Shanghai, China; Harbin, China; Chongqing, China; etc.	Spring, Summer, Autumn, and Winter	Outdoor thermal comfort	Not directly
[15]	Cheng. H., Han. Y., Park. C.	Urban space	Seoul, South Korea	Summer	Green infrastructure types	Not directly
[16]	Qiao. L.	Public space	Guangzhou, China	Autumn	Vertical greening	Yes

**Table 2:** Validation accuracy performance of AT in ENVI-met model simulations.

Settings	R^2^/RMSE (AT)	R^2^/RMSE (RH)	R^2^/RMSE (WS)
Grid size of 2 × 2 m^2^	0.944/1.77	0.892/1.23	0.872/0.22
Grid size of 4 × 4 m^2^	0.938/1.88	0.756/1.88	0.641/0.40
Grid size of 8 × 8 m^2^	0.932/1.96	0.643/2.54	0.203/0.81

**Table 3:** Setting depiction.

Parameters	Experiment Groups		
Original	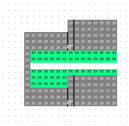	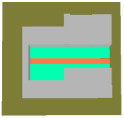	S1 Grassland:85% Cray brick:15%
Space layout	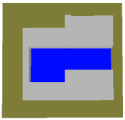	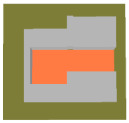	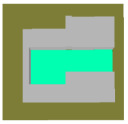
	S2	S3	S4
	Water:100%	Cray brick:100%	Grassland:100%
Scatter layout 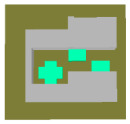	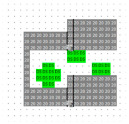	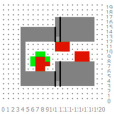	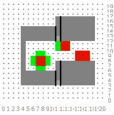
	S5	S6	S7
	DT:0%	DT:20%	DT:40%
	GT:100%	GT:80%	GT:60%
	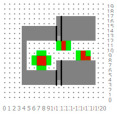	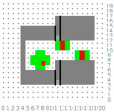	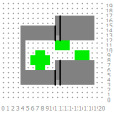
	S8	S9	S10
	DT:60%	DT:80%	DT:100%
	GT:40%	GT:20%	GT:00%
Array layout 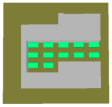	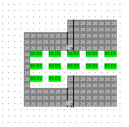	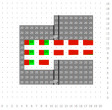	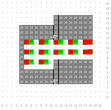
	S11	S12	S13
	DT:0%	DT:20%	DT:40%
	GT:100%	GT:80%	GT:60%
	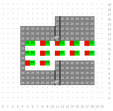	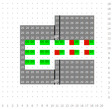	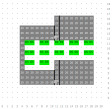
	S14	S15	S16
	DT:60%	DT:80%	DT:100%
	GT:40%	GT:20%	GT:00%

**Table 4:** Three-way ANOVA results.

Source	Sum of Squares	df	Mean Square	F-Value	*p*-Value	R^2^
Intercept	27,197.386	1	27,197.386	2,212,895.158	0.000 ***	0.982
Season	5469.642	3	1823.214	148,344.443	0.000 ***	
Planting method	0.267	1	0.267	21.721	0.000 ***	
Tree species ratio	1.112	5	0.222	18.091	0.000 ***	
Error	0.467	38	0.012	-	NaN	

*** indicates statistical significance at *p* < 0.001.

## Data Availability

The original contributions presented in this study are included in the article. Further inquiries can be directed to the corresponding author.
